# *Morus alba* and active compound oxyresveratrol exert anti-inflammatory activity via inhibition of leukocyte migration involving MEK/ERK signaling

**DOI:** 10.1186/1472-6882-13-45

**Published:** 2013-02-23

**Authors:** Yi-Ching Chen, Yin-Jing Tien, Chun-Houh Chen, Francesca N Beltran, Evangeline C Amor, Ran-Juh Wang, Den-Jen Wu, Clément Mettling, Yea-Lih Lin, Wen-Chin Yang

**Affiliations:** 1 Agricultural Biotechnology Research Center, Academia Sinica, 128, Sec. 2, Academia Road, Nankang, Taipei 11501, Taiwan; 2 Institute of Statistical Science, Academia Sinica, Taipei, Taiwan; 3 Institute of Chemistry, College of Science, University of the Philippines, Quezon City, Philippines; 4 Miaoli District Agricultural Research and Extension Station, Council of Agriculture, Miaoli, Taiwan; 5 Institut de Génétique Humaine (CNRS UPR-1142), Montpellier, France; 6 Department of Life Sciences, National Chung Hsing University, Taichung 402, Taiwan; 7 Institute of Pharmacology, Yang-Ming University, Taipei, 112, Taiwan; 8 Institute of Zoology, National Taiwan University, Taipei, 106, Taiwan

**Keywords:** Chemotaxis, CXCR4, *Morus*, Phytochemistry and T-cells

## Abstract

**Background:**

*Morus alba* has long been used in traditional Chinese medicine to treat inflammatory diseases; however, the scientific basis for such usage and the mechanism of action are not well understood. This study investigated the action of *M. alba* on leukocyte migration, one key step in inflammation.

**Methods:**

Gas chromatography-mass spectrometry (GC-MS) and cluster analyses of supercritical CO_2_ extracts of three *Morus* species were performed for chemotaxonomy-aided plant authentication. Phytochemistry and CXCR4-mediated chemotaxis assays were used to characterize the chemical and biological properties of *M. alba* and its active compound, oxyresveratrol. fluorescence-activated cell sorting (FACS) and Western blot analyses were conducted to determine the mode of action of oxyresveratrol.

**Results:**

Chemotaxonomy was used to help authenticate *M. alba.* Chemotaxis-based isolation identified oxyresveratrol as an active component in *M. alba*. Phytochemical and chemotaxis assays showed that the crude extract, ethyl acetate fraction and oxyresveratrol from *M. alba* suppressed cell migration of Jurkat T cells in response to SDF-1. Mechanistic study indicated that oxyresveratrol diminished CXCR4-mediated T-cell migration via inhibition of the MEK/ERK signaling cascade.

**Conclusions:**

A combination of GC-MS and cluster analysis techniques are applicable for authentication of the *Morus* species. Anti-inflammatory benefits of *M. alba* and its active compound, oxyresveratrol, may involve the inhibition of CXCR-4-mediated chemotaxis and MEK/ERK pathway in T and other immune cells.

## Background

The genus *Morus* from the Moraceae family consists of 10–16 species of deciduous trees that are distributed worldwide [1]. Different parts of the *Morus* plants such as leaves, fruit, branches, bark, root, and shoot have been used as food and herbal medicine in China for over 1900 years [2]. In Taiwan, *M. alba*, commonly known as white mulberry, is possibly the *Morus* species most frequently used in traditional Chinese medicine although it is sometimes used interchangeably with *M. atropurpurea* and *M. bombycis*. As all three species have similar morphological characteristics and habitat preferences they are frequently misidentified. *M. alba*, the most extensively studied species, has been reported to have anti-hyperlipidemic [3], anti-hypertensive [4,5], anti-hyperglycemic [2,6]; Hansawasdi, 2006; [7], anti-microbial [8-12], anti-allergic [13], anti-inflammatory [14,15], hepatoprotective [16,17], neuroprotective [18], immuno-modulatory [19], and anti-venom activities [20].

Photochemical studies have identified alkaloids, flavonoids, flavones, flavanones, stilbenes, benzophenones, coumarin derivatives and terpenoids in *Morus* species [5,21-37]. These compounds are likely responsible for the bioactivities of the *Morus* plants. Among them, the stilbenes, oxyresveratrol and resveratrol, were reported present in the *Morus* plants and demonstrated antioxidant activity [38]. Oxyresveratrol inhibited nitrogen oxide (NO) production, inducible NO synthase (iNOS) expression, prostaglandin E2 (PGE_2_) production, and activation of nuclear factor kappa-light-chain enhancer of activated B cells (NFκB) in macrophages [39]; and consistently reduced edema induced by carrageenan in a mouse model [39]. The above data suggest the involvement of oyxresveratrol in suppression of the inflammatory process. Nevertheless, the role of oxyresveratrol in the regulation of leukocyte migration has not been studied.

Migration of leucocytes from blood vessels to the flamed sites is a fundamental feature of inflammation. Chemokines and chemokine receptors can orchestrate leukocyte migration, also termed chemotaxis [40]. In addition to its physiological functions, chemotaxis is implicated in inflammation and disease pathogenesis [41]. Therefore, chemotaxis has been proposed as a key target of anti-inflammatory drugs [42]. CXCR4, a G-protein-linked transmembrane receptor, is expressed in all leukocytes, blastocysts and a variety of cancer cells [43]. SDF-1 (CXCL12) is a natural ligand of CXCR4. After binding to SDF-1, CXCR4 triggers a signaling cascade which includes the activation of kinases (FAK, PI3K, ERK, JAK, and TYK) and downstream molecules (NFκB and STAT) and, eventually regulates chemotaxis, locomotion, and adhesion [43].

Plants provide an extraordinary source of lead compounds for a myriad of disorders including inflammation. In this study, we investigated the role of *M. alba* in leukocyte migration, a key step in inflammation. As *M. alba* is easily misidentified due to its close resemblance to other *Morus* plants, we first devised a chemotaxonomic strategy involving GC-MS analysis and cluster analysis of the chemical profiles to differentiate between the *Morus* species. Next, the anti-chemotactic activities of *M. alba* and its active compound, oxyresveratrol, were examined in Jurkat T cells. Finally, the mode of action of *M. alba* and its active compound, oxyresveratrol, was investigated.

## Methods

### Reagents

High performance liquid chromatography (HPLC) grade solvents, acetonitrile (ACN), dichloromethane, butanol, methanol and ethyl acetate were purchased from Avantor Performance Materials (NJ, USA). Trifluoroacetic acid (TFA), methanol, dimethyl sulfoxide (DMSO), resveratrol and oxyresveratrol were purchased from Sigmal (MO, USA). RPMI 1640 medium, PSQ solution (penicillin, streptomycin and glutamine), sodium pyruvate, non-essential amino acids and HEPES were purchased from Gibco (CA, USA). αCXCR4, FITC-conjugated secondary antibody (Life Technologies, NY, USA) and SDF-1 (R&D systems, MN, USA) were purchased. WST-1 reagent was purchased from Roche (Mannheim, Germany). Antibodies against mitogen-activated protein kinases (MAPKs) and their phosphorylated proteins were purchased from Cell Signaling Technology (MA, USA).

### Supercritical liquid extraction and GC-MS analysis of plants

*M. alba*, *M. atropurpurea* and *M. bombycis* were collected and authenticated by Dr Ran-Juh Wang from the Miaoli District Agricultural Research and Extension Station (MDARES), Miaoli County, Taiwan, in 2010. Their voucher specimens were deposited as No. 00083241, No. 00082146, and No. 00083303, respectively, at the MDARES herbarium. Branches of the plants were air dried and pulverized. Five grams of plant samples were extracted with carbon dioxide using the supercritical fluid extractor SFX System 1220R (ISCO, NE, USA), followed by GC-MS analysis using a Trace gas chromatograph interfaced to a Polaris Q mass spectrometer (Thermo Finnigan, Hertfordshire, UK) in EI mode (70 eV) as previously described [44].

### Extract preparation, HPLC and compound identification

Branches of *Morus* plants were pulverized and extracted with methanol (2 × 3 L). The methanol extracts were evaporated by rotary evaporator, yielding 1 g of crude extracts. The *M. alba* crude extract was re-suspended with water (150 mL), followed by sequential extraction with ethyl acetate (9 × 150 mL) and butanol (6 ×150 mL). Crude extracts, fractions, and oxyresveratrol were analyzed using an Agilent 1100 Series HPLC system. HPLC was performed on a Luna C18 column (Phenomenex, CA, USA) at a flow rate of 0.5 ml/min and 25°C with a photodiode detector at 254 nm. The solvent gradient for HPLC was set in the following sequence, 10% to 50% of B for 0–50 min, 50% of B for 50–65 min, 50 to 100% of B for 65–70 min, and 100% of B for 70 to 95 min. Solvents A and B were 0.05% TFA/H_2_O and 0.05% TFA/CAN, respectively. The identity of oxyresveratrol with a purity of 97% from *M. alba* was confirmed by comparing its spectroscopic data with previously published data [45,46].

### Chemotaxis assay and WST-1 assay

Jurkat cells E6.1 (ATCC No. TIB-152), a leukemic T cell line, were cultured in complete medium as described [47]. The cells (1 × 10^6^/mL) were pre-treated with crude extract, fractions, or compounds of *M. alba* for 24 h in 1% FBS medium. The cells were transferred into a transwell insert (Millipore, USA) and put into a 24-well plate where SDF-1 or vehicle (PBS) was added to the 1% FBS medium. After 4 h, the cells that migrated to the bottom of the 24-well plate were counted and photographed. The migration index (MI) was defined below. MI (%) = 100 × (number of drug-treated cells migrating toward SDF-1 minus number of drug-treated cells migrating toward PBS)/(number of vehicle-treated cells migrating toward SDF-1 minus number of vehicle-treated cells migrating toward PBS) [47]. For WST-1 assay, Jurkat cells (1 × 10^6^/mL) were incubated with the crude extract, fractions and compound for 24 h. After PBS washing, the cells were incubated with WST-1 for 1 h and measured at 440 nm using the BioTek ELISA reader (VT, USA).

### Western blot and FACS analysis

Jurkat cells were pre-treated with vehicle and compounds at the indicated concentrations for 1 h. For Western blot, the cells were stimulated with SDF-1 for the indicated time. Total lysates underwent sodium dodecyl sulfate polyacrylamide gel electrophoresis and blotting with the indicated antibodies, followed by ECL visualization. To determine the CXCR4 expression level, the cells were stained with αCXCR4 and secondary antibody and underwent FACS analysis using FlowJo software.

### Statistical analysis

Data from three experiments or more are presented as mean ± standard deviation. Comparisons between experimental groups and control were made using ANOVA. *P* values (*) less than 0.05 were considered statistically significant.

## Results

### GC-MS chromatograms and cluster analysis of the chemical profiles of *Morus* species

**Figure 1:**
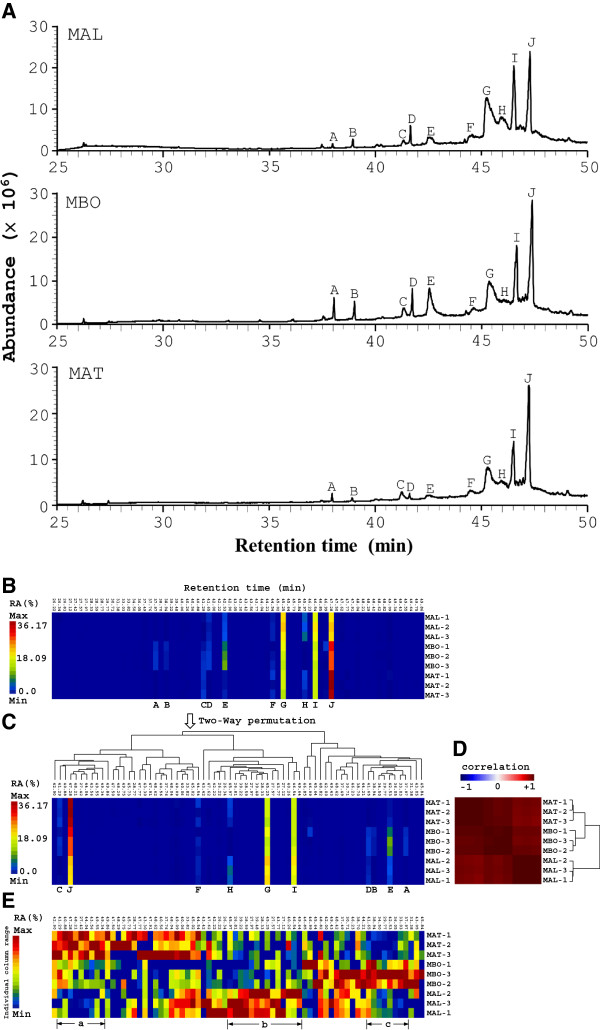
**GC-MS profiles and cluster analysis of the three *Morus *species. **(**A**) Branches of *M. alba *(MAL), *M. atropurpurea* (MAT), and *M. bombycis *(MBO) underwent supercritical CO_2_ extraction and GC-MS analysis at a dose of 10 mg/ml. One representative GC-MS chromatogram from 3 batches of each plant extract is shown. The sizes of the various GC-MS peaks are measured as percent relative area (RA (%)) of the largest GC-MS peak in the chromatogram. (**B**) Matrix visualization with hierarchical cluster trees (HCT) for GC-MS profiles of the *Morus *species. A representative GC-MS profile of the 3 *Morus *species from Figure 1 is displayed as a matrix map with 9 rows, each representing one sample, and 70 columns, each representing one retention time. A rainbow spectrum was used to color code the size of the chromatographic peaks (expressed in percent relative area, RA (%)) in the whole matrix. Red denotes the largest peak (maximum RA (%)) and blue denotes the smallest peak (minimum RA (%)). (**C**) Two-way permuted data matrix of (B). The tree structure above the data matrix represents average linkage hierarchical cluster tree (HCT) for 70 different retention times. (**D**) Map of correlation matrix for the 9 samples (three samples per species). A bi-directional blue–white–red spectrum is used to denote negative-to-positive correlation. The tree structure besides the correlation matrix represents average linkage HCT for 9 samples. (**E**) Identical data matrix map as in (**B**) except each column is colored using a rainbow color spectrum scaled individually for that particular column instead of a rainbow color spectrum scaled for all columns in the matrix.

Correct authentication of medicinal plants is an essential prerequisite to ensure reproducible quality and efficacy of herbal medicine. *M. alba*, *M. atropurpurea* and *M. bombycis* are all common in Taiwan. However, their dried samples and products are frequently indistinguishable. For this purpose, a combination of GC-MS and cluster analysis tools were used to rapidly distinguish the three *Morus* species. First, GC-MS analysis of the supercritical CO_2_ extracts was used to profile the chemical compositions of the three species (Figure 1A). Peaks A to J are shown in Figure 1. As previously described [48,49], cluster analysis tools, such as hierarchical cluster trees (HCT), principal component analysis (PCA) and multidimensional scaling (MDS), were next used to analyze the inter- and intra-species chemical variations. In this work, we adopted HCT with matrix visualization (MV) in generalized association plots (GAP) [50,51] to compare the similarities and differences in the chemical profiles of the *Morus* extracts (Figure 1A). Figure 1B shows the matrix visualization of the sizes of the GC-MS peaks in percent relative areas [RA (%); area of a GC-MS peak normalized against the area of the largest peak in the chromatogram, presented as a percentage] of 9 samples (3 samples of each species) with 70 retention times between 26.22 min and 49.86 min. A rainbow spectrum was used to color code the sizes of the chromatographic peaks with red denoting the largest peak [maximum RA(%)] and blue denoting the smallest peak [minimum RA(%)]. Each row in Figure 1B represents one sample while each column represents one particular retention time. Average linkage hierarchical cluster trees (HTCs) for retention time and for samples were then built on corresponding correlation matrices for retention time and samples, respectively. Figure 1C is obtained from the two-way permutation according to the orders of terminal nodes in HCTs for retention time and samples in order to obtain sample cluster structure and retention time grouping pattern [51]. In Figure 1D, a bi-directional blue-white-red spectrum is used to denote negative-to-positive correlation. The most profound observation to be noted in Figure 1D was that *M. atropurpurea* and *M. bombycis* samples form a larger group with high positive within-group correlation (in dark red) while *M. alba* samples form a separate cluster.

By converting Figure 1C to Figure 1E using individually ranged color spectra for each retention time so that between-variant structure within each retention time (column) can be clearly depicted, we found that the combination of matrix visualization for GC-MS chemical profiles with hierarchical cluster trees using GAP was a fast and reliable chemotaxonomical method to assist in the identification of the three *Morus* species. In Figure 1E, the GC-MS peaks of *M. alba* (MAL), *M. atropurpurea* (MAT) and *M. bombycis* (MBO) are relatively larger than others with retention time in groups a, b and c, respectively. By selecting the components of groups a, b, and c in Figure 1E and significant relative peak areas in Figure 1C, we identified peaks J, G, I, and E (Figure 1C) as useful parameters to distinguish between the three species.

Overall, the combination of GC-MS and cluster tools is feasible and suitable for determining the taxonomy of the *Morus* species.

### HPLC profiles of the three *Morus* plant extracts

**Figure 2:**
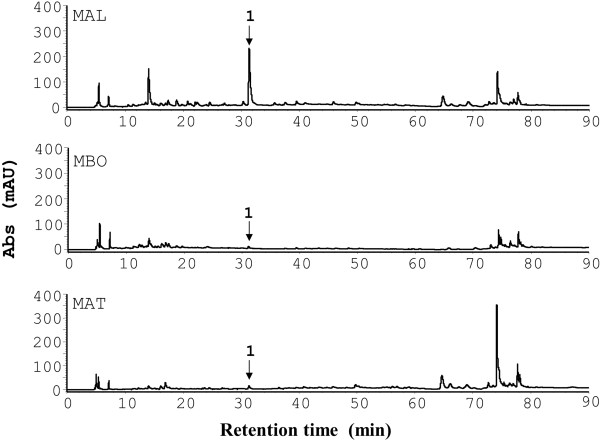
**HPLC profiles of the crude extracts of the three *Morus *species. **Methanol crude extracts of each species at a dose of 25 mg/ml were analyzed using an RP-18 HPLC column and detected with a UV detector at 254 nm. Peak 1 (oxyresveratrol) is indicated.

**Figure 3:**
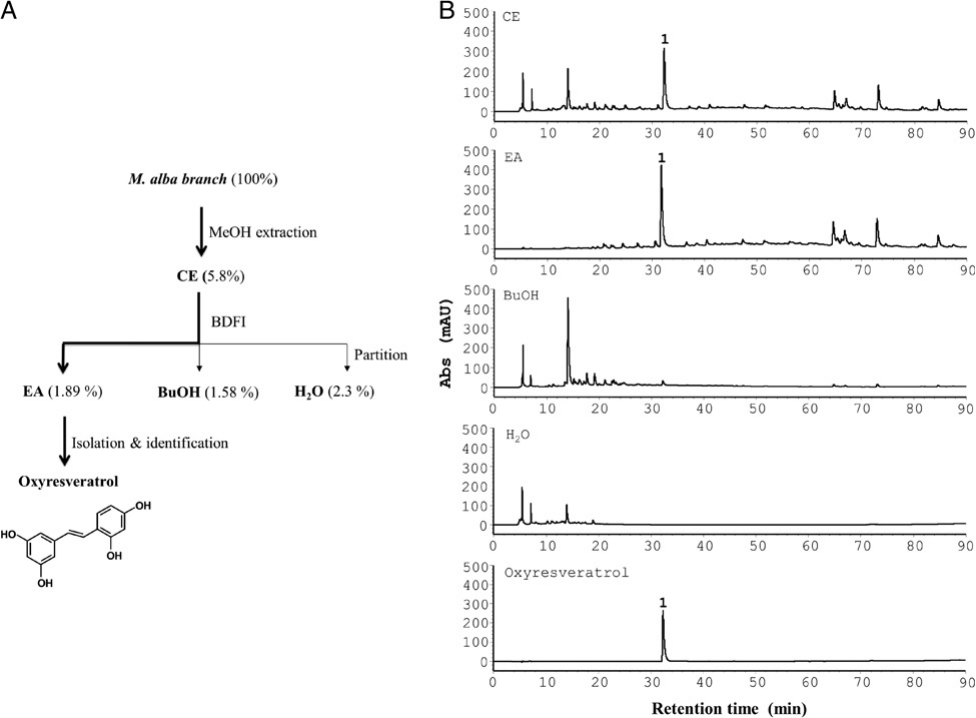
**Chemotaxis-based chemical analysis of *M. alba*. **(**A**) Flow chart of the bioactivity-directed fractionation and isolation (BDFI) analysis. A *M. alba *branch was ground and extracted with methanol (MeOH). The crude extract (CE) was tested for bioactivity using chemotaxis assay. The same crude extract was sequentially partitioned with ethyl acetate (EA), butanol (BuOH and water (H_2_O). The bioactivity of the 3 fractions was further tested. Finally, active compound, oxyresveratrol, was purified from the active EA fraction. The dry weights of the crude extract, fractions and compound of *M. alba* are indicated as percentages of the dry weight of the raw plant material. (**B**) HPLC profiles of methanol crude extract (CE), ethyl acetate fraction (EA), butanol fraction (BuOH), water fraction (H_2_O) and oxyresveratrol with UV detection at 254 nm. Peak 1 is identified as oxyresveratrol.

Having authenticated the *M. alba*, *M. atropurpurea*, and *M. bombycis* branches, we next sought to characterize their chemical properties. HPLC is a useful method for analysis and comparison of chemical fingerprints of plant extracts. HPLC analysis showed similar and distinct peaks among the 3 *Morus* species (Figure 2). The peaks at the retention times, 14.0, 31.3, 64.7, 74.0 and 77.6 min, appeared in all three species despite the difference in the relative amount of the peaks. Of note, peak 1 at the retention time of 31.3 min was present in the crude extracts of the three *Morus* species. In contrast, *M. alba* had a more abundant peak 1 than *M. atropurpurea* and *M. bombycis*. Peak 1 was isolated from the *M. alba* extract and identified as oxyresveratrol as shown in Figure 3. The NMR data (Additional file 1: Table S1), UV spectra data (Additional file 1: Figure S1A) and MS data (Additional file 1: Figure S1B) of oxyresveratrol in the study were analyzed and confirmed with those described elsewhere [45,46].

### Characterization of *M. alba* extract using a combination of phytochemical and chemotaxis assays

*M. alba* has long been used as anti-inflammatory medicine. To further understand the anti-inflammtory activity of *M. alba*, a chemotaxis-guided fractionation and isolation strategy was adopted (Figure 3A). HPLC analysis of the crude extract, fractions and oxyresveratrol, an active compound, of *M. alba* showed that oxyresveratrol (peak 1) was present in the crude extract (CE, Figure 3B) and ethyl acetate fraction (EA, Figure 3B) of *M. alba*; however, oxyresveratrol was not detectable in the butanol (BuOH, Figure 3B) and water (H_2_O, Figure 3B) fractions of *M. alba*.

**Figure 4:**
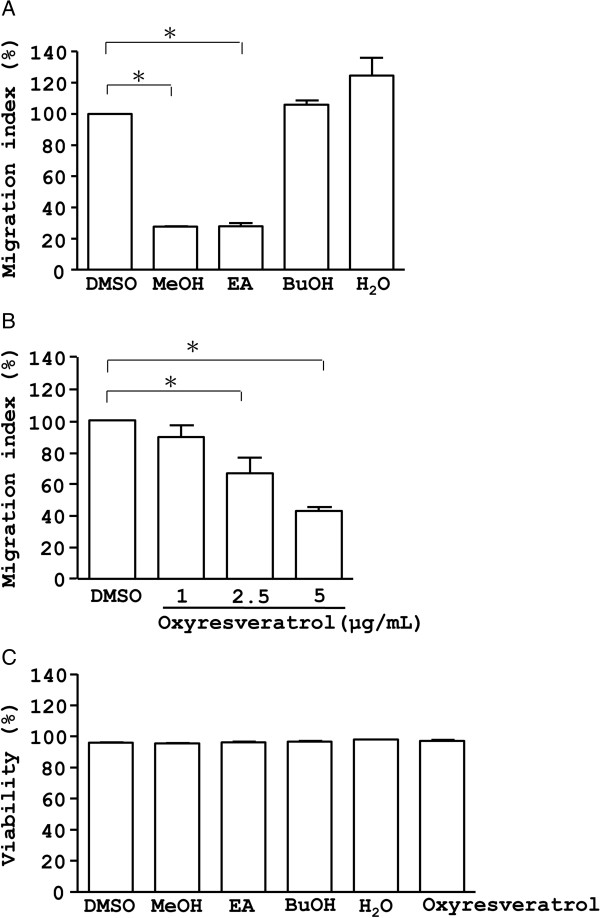
**Effect of crude extract, fractions and compound of *M. alba *on chemotaxis and cell viability. **(**A**) Jurkat cells were pre-treated with vehicle (DMSO), the crude extract (CE, 10 μg/ml), butanol fraction (BuOH, 4 μg/ml), ethyl acetate fraction (EA, 4 μg/ml) and water fraction (H_2_O, 10 μg/ml) of *M. alba *for 24 h. The cells were treated with PBS and SDF-1, respectively, for an additional 4 h in transwell microplates. The number of cells in the bottom well was counted. Cell migration is indicated as migration index (%), as defined in Materials and methods. (**B** &**C**) Jurkat cells were pre-treated with oxyresveratrol at 1, 2.5 and 5 μg/ml. The cells underwent chemotaxis assays (**B**) or WST-1 test (**C**) for cell viability. *P *(*) < 0.05 is considered statistically significant.

Examination of the effect of the crude extract, fractions and oxyresveratrol of *M. alba* on CXCR4-mediated chemotaxis further showed that the *M. alba* crude extract and the ethyl acetate fraction inhibited chemotaxis in Jurkat T cells, key players in immune response (Figure 4A). In contrast, the butanol and water fractions of *M. alba* did not inhibit CXCR4-mediated chemotaxis (Figure 4A). Consistently, oxyresveratrol dose-dependently inhibited CXCR4-mediated chemotaxis in T cells (Figure 4B). This inhibition by the crude extract, fractions and oxyresveratrol of *M. alba* was not due to cytotoxicity because treated cells showed high viability (Figure 4C). Overall, the data suggest that *M. alba* and oxyresveratrol suppress inflammation via inhibition of leukocyte migration.

### Mechanistic study of oxyresveratrol in CXCR4-mediated chemotaxis

**Figure 5:**
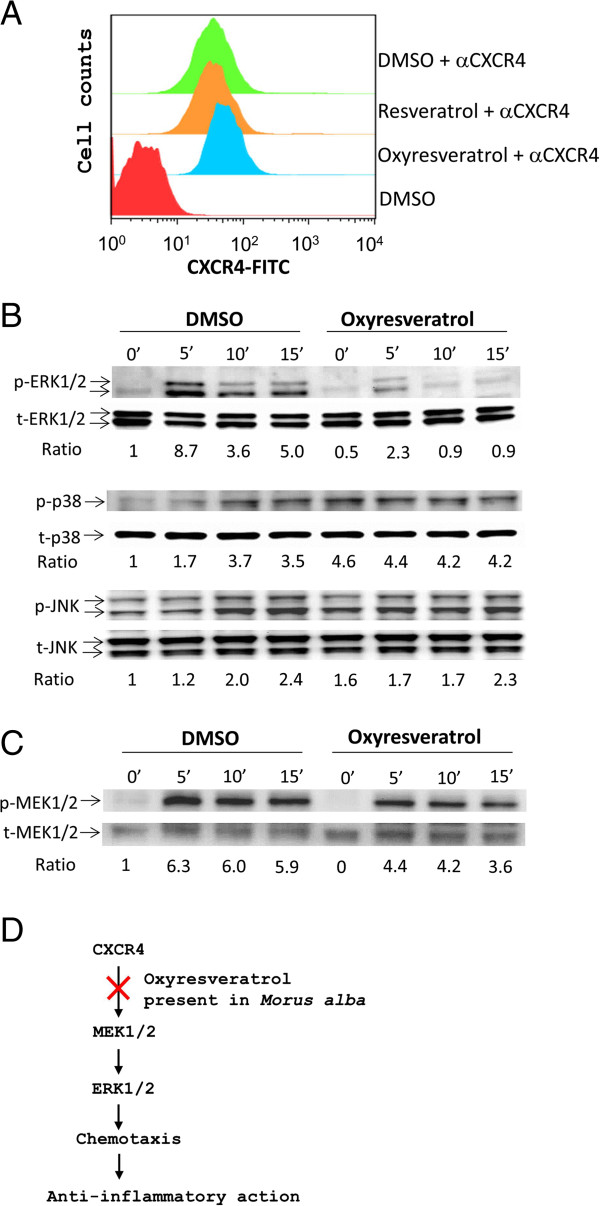
**Effect of oxyresveratrol on CXCR4 signaling. **(**A**) Jurkat cells were pre-treated with oxyresveratrol (2.5 μg/ml), resveratrol (2.5 μg/ml) or DMSO vehicle for 1 h at 37°C. After extensive washing, the cells were stained with αCXCR4 (1 μg/ml) and FITC-conjugated secondary antibody (1 μg/ml) or not. The expression level of CXCR4 on the cell membrane is shown in the histogram. Data are representative examples of 3 experiments. (**B** &**C**) The cells, pre-treated with oxyresveratrol or DMSO, were stimulated with SDF-1 (100 ng/ml) for 0 to 15 min. Cell lysates were analyzed by Western blot with primary antibodies (1 μg/ml) against MAPKs (**B**), MEK1/2 (**C**) or their phosphorylated forms (**B **&**C**) plus secondary antibodies (0.3 μg/ml). The ratio was obtained by normalizing the signal of the phosphorylated protein to that of the total protein. (**D**) A schematic model describing the mechanism by which oxyresveratrol present in *M. alba *can inhibit inflammation.

To dissect the mechanism by which oxyresveratrol inhibited leukocyte migration, we first examined whether oxyresveratrol could down-regulate the level of cell surface expression of the chemokine receptor CXCR4. Jurkat T cells were treated with oxyresveratrol and the CXCR4 expression level on T cells was measured using FACS analysis. FACS data showed no difference in the surface expression of CXCR4 on cells treated with oxyresveratrol, resveratrol, or DMSO vehicle (Figure 5A), suggesting that oxyresveratrol does not act at the chemokine receptor level.

Next, we investigated the impact of oxyresveratrol on the CXCR4 downstream signaling molecules. MAPKs are known to function downstream of chemokine receptors [47]; therefore, we examined whether oxyresveratrol influenced MAPKs in the CXCR4 pathway. Western blot data showed that oxyresveratrol inhibited SDF-1-mediated phosphorylation of ERK1/2 kinases (Figure 5B). Conversely, it enhanced the SDF-1-mediated phosphorylation of p38 (Figure 5B). However, oxyresveratrol did not appear to affect SDF-1-mediated JNK phosphorylation (Figure 5B). Knowing that oxyresveratrol inhibited the activation of ERK1/2, we further examined the effect of oxyresveratrol on the upstream activators of ERK1/2 kinases, MEK1/2 kinases. Accordingly, it suppressed the phosphorylation of MEK1/2 kinases in the CXCR4 pathway (Figure 5C). These data demonstrated that oxyresveratrol in *M. alba* suppresses CXCR4-mediated chemotaxis via inactivation of the ERK signaling pathway. The data on Jurkat cells can be confirmed with normal leukocytes. Moreover, overall data suggest that anti-inflammatory herb, *M. alba*, and its oxyresveratrol suppress inflammation via inhibition of leukocyte migration involving the MEK/ERK pathway (Figure 5D).

## Discussion and conclusions

Correct identification and authentication of plants are absolutely necessary for batch consistency and therapeutic efficacy of botanical medicines. *M. alba* is used in traditional Chinese medicine for treatment of inflammatory disorders. However, *M. alba* is sometimes used interchangeably with, or confused with other *Morus* species such as *M. atropurpurea* and *M. bombycis*. A standard method of identification of *M. alba* versus *M. atropurpurea* or *M. bombycis* has not been established, and the similarities/differences between the chemical and biological properties of these three species have not been studied. In this work, we first used chemotaxonomic methods to differentiate *M. alba* from the other two species. Spectroscopic methods were used to analyze the chemical fingerprint of crude extracts, fractions, and compounds of *M. alba*. Combined photochemistry and chemotaxis assays were then used for identification and anti-inflammatory study of *M. alba* and oxyresveratrol.

Chemokines and their receptors are involved in numerous diseases and have roles in inflammation and infection. Consequently, the antagonists and inhibitors of the chemokines and their receptors have become potential drug targets for inflammatory diseases [41]. *M. alba* has been long used as anti-inflammatory remedy in China. Nevertheless, little is known about its role in leukocyte migration, a central process linked to inflammation, infection and disease pathogenesis. Here, we demonstrated that *M. alba* can suppress leukocyte migration triggered by CXCR4 (Figure 4). Unexpectedly, bioassay-guided isolation and identification yielded oxyresveratrol as the major active component of the bioactive ethyl acetate fraction of *M. alba* (Figure 3). *M. alba* possessed a higher abundance of oxyresveratrol than the other *Morus* plants (Figure 2). The evidence presented here that *M. alba* and its active component, oxyresveratrol, suppress CXCR4-mediated leukocyte migration supports the traditional use of *M. alba* as an anti-inflammatory medicine.

Oxyresveratrol has been previously reported to exert anti-inflammatory activity through inhibition of iNOS/NO production, PGE2 synthesis and NFκB activation [39]; and was reported to reduce carageenan-induced paw edema in rats likely through inhibition of iNOS expression [39]. We have also observed oxyresveratrol to be a more potent inhibitor of leukocyte migration than resveratrol (Figure 4B and data not shown). Our data on the difference in chemotactic action between resveratrol and oxyresveratrol are in good agreement with a previous publication indicating that resveratrol does not inhibit chemotaxis [47]. Thus, the OH functional group in oxyresveratrol appears to be responsible for its anti-chemotactic activity. Furthermore, oxyresveratrol inhibited the activation of the MEK/ERK pathway (Figure 5), suggesting a possible mechanism by which *M. alba* and oxyresveratrol inhibit inflammation.

Following engagement, chemokine receptors induce an activation of G proteins, tyrosine kinases, serine/threonine kinases and phospholipases, leading to cell migration [52,53]. Our results showed that oxyresveratrol, isolated from *M. alba*, inhibited the activation of MEK/ERK kinases, a serine/threonine kinase family, mediated by CXCR4 in T-cells (Figure 5). However, oxyresveratrol did not affecte the expression level of CXCR4 receptor (Figure 4A). These findings suggest that unlike the receptor antagonists, oxyresveratrol targets the intraceullar proteins downstream of the chemokine receptors and may be used as alternative inhibitors of chemokine signaling. Consistently, MAPKs are known to modulate inflammatory responses and are thought to be attractive molecular targets for anti-inflammatory therapy [54]. The manifestation of inactivation of MEK/ERK pathway by oxyresveratrol makes it extremely interesting potential natural anti-inflammatory remedy.

In conclusion, we combined phytochemical and chemotaxis techniques to study the anti-chemotactic role of the *Morus* plants. We demonstrated that *M. alba* and its active compound, oxyresveratrol, suppress inflammation via inhibition of leukocyte chemotaxis. Mechanistic studies showed that oxyresveratrol inhibits CXCR4-mediated leukocyte migration via inactivation of the MEK/ERK pathway downstream of the CXCR4 receptor. These findings support the claims of the benefits of *M. alba* purported in traditional Chinese medicine and suggest the possible use of the active compound oxyresveratrol as an anti-inflammation therapy.

## Abbreviations

FACS: Fluorescence-activated cell sorting; GC-MS: Gas chromatography-mass spectrometry; HPLC: High performance liquid chromatography; iNOS: Inducible NO synthase; NFκB: Nuclear factor kappa-light-chain enhancer of activated B cells; NO: Nitrogen oxide; PGE2: Prostaglandin E2

## Competing interest

The authors declare that they have no competing interests.

## Authors’ contributions

YLL, CM and WCY conceived the study. YCC, YJT and FNB carried out the experiments. CHC and ECA helped YCC, YJT and FNB in carrying out the experiments. RJW and DJW participated in designing the experiments and plant authentication. WCY wrote the manuscript. All authors read and approved the final manuscript.

## Pre-publication history

The pre-publication history for this paper can be accessed here:

http://www.biomedcentral.com/1472-6882/13/45/prepub

## Supplementary Material

Additional file 1: Table S1 ^1^H and ^13^C NMR data on oxyresveratrol dissolved in CD_3_COCD_3_. **Figure S1. **Ultraviolet (UV) spectra and mass spectrometry data of oxyresveratrol present in the crude extract and ethyl acetate fraction of *Morus alba *and oxyresveratrol. (A) The crude extract (CE) and ethyl acetate (EA) fraction and resveratrol were subjected to high performance liquid chromatography (HPLC) and detected with a diode array detector at 254 nm as described in the Materials and methods section. The UV spectra of resveratrol (peak 1) are indicated. Peak 1 corresponds to the same peak as Figure B. (B) Electrospray ionization mass spectrometry (ESI-MS) spectra of oxyresveratrol present in the crude extract and ethyl acetate fraction of *M. alba *and oxyresveratrol. The crude extract and ethyl acetate fraction of *M. alba *and oxyresveratrol were subjected to HPLC-ESI-MS. The MS scans were performed in negative ion mode (m/z 200 to m/z 400). Peaks 1 (31.3 min) of the crude extract (CE) and ethyl acetate fraction (EA) of *M. alba *and oxyresveratrol showed ion signals at m/z 243. Peak 1 corresponds to the same peak as Figure 3B. Click here for file
