# Supplementary material for: Morus alba and active compound oxyresveratrol exert anti-inflammatory activity via inhibition of leukocyte migration involving MEK/ERK signaling
Source: BMC Complement Altern Med. 2013 Feb 23;13:45. doi: 10.1186/1472-6882-13-45 (PMC3639811; doi:10.1186/1472-6882-13-45)
Supplement: Additional file 1: Table S1 — 1H and 13C NMR data on oxyresveratrol dissolved in CD3COCD3. Figure S1. Ultraviolet (UV) spectra and mass spectrometry data of oxyresveratrol present in the crude extract and ethyl acetate fraction of Morus alba and oxyresveratrol. (A) The crude extract (CE) and ethyl acetate (EA) fraction and resveratrol were subjected to high performance liquid chromatography (HPLC) and detected with a diode array detector at 254 nm as described in the Materials and methods section. The UV spectra of resveratrol (peak 1) are indicated. Peak 1 corresponds to the same peak as Figure B. (B) Electrospray ionization mass spectrometry (ESI-MS) spectra of oxyresveratrol present in the crude extract and ethyl acetate fraction of M. alba and oxyresveratrol. The crude extract and ethyl acetate fraction of M. alba and oxyresveratrol were subjected to HPLC-ESI-MS. The MS scans were performed in negative ion mode (m/z 200 to m/z 400). Peaks 1 (31.3 min) of the crude extract (CE) and ethyl acetate fraction (EA) of M. alba and oxyresveratrol showed ion signals at m/z 243. Peak 1 corresponds to the same peak as Figure 3B. [file 1472-6882-13-45-S1.doc]

***Morus alba* and active compound oxyresveratrol exert anti-inflammatory activity via inhibition of leukocyte migration involving MEK/ERK signaling**

Yi-Ching Chen^1*^, Yin-Jing Tien^2*^, Chun-Houh Chen^2^, Francesca N. Beltran^3^, Evangeline C. Amor^3^, Ran-Juh Wang^4^, Den-Jen Wu^4^, Clément Mettling^5^, Yea-Lih Lin^5^ and Wen-Chin Yang^1,6,7,8§^

**Supplemental information**

**Supplemental Table 1.** ^1^H and ^13^C NMR data on oxyresveratrol dissolved in CD_3_COCD_3_.

| H/C | δ_H_,multiplicity (*J*/Hz) | δ_C_,multiplicity(*J*/Hz) |
| --- | --- | --- |
| 1 |  | 141.6 s |
| 2 | 6.5 dd (0.9) | 105.3 d |
| 3 |  | 159.5 d |
| 4 | 6.22 d (2.1) | 102.1 d |
| 5 |  | 159.5 d |
| 6 | 6.51 d (0.9) | 105.3 d |
| 7 | 7.32 d (8.1) | 124.2 d |
| 8 | 6.88 d (9.9) | 126.2 d |
| 1’ |  | 117.2 s |
| 2’ |  | 156.8 s |
| 3’ | 6.43 d (1.2) | 103.5 d |
| 4’ |  | 159.0 s |
| 5’ | 6.36-8 dd (0.9) | 108.3 d |
| 6’ |  | 128.2 d |
| OH |  |  |
|  | 8.15 s, H |  |
|  | 8.36 s, H |  |
|  | 8.55 s, 2H |  |

**Supplemental Fig. 1.** Ultraviolet (UV) spectra and mass spectrometry data of oxyresveratrol present in the crude extract and ethyl acetate fraction of *Morus alba* and oxyresveratrol. (A) The crude extract (CE) and ethyl acetate (EA) fraction and resveratrol were subjected to high performance liquid chromatography (HPLC) and detected with a diode array detector at 254 nm as described in the Materials and methods section. The UV spectra of resveratrol (peak **1**) are indicated. Peak **1** corresponds to the same peak as Figure 3B. (B) Electrospray ionization mass spectrometry (ESI-MS) spectra of oxyresveratrol present in the crude extract and ethyl acetate fraction of *M. alba* and oxyresveratrol. The crude extract and ethyl acetate fraction of *M. alba* and oxyresveratrol were subjected to HPLC-ESI-MS. The MS scans were performed in negative ion mode (m/z 200 to m/z 400). Peaks **1** (31.3 min) of the crude extract (CE) and ethyl acetate fraction (EA) of *M. alba* and oxyresveratrol showed ion signals at m/z 243. Peak **1** corresponds to the same peak as Figure 3B.
